# Determinants of the intention to seek psychotherapeutic consultation at work - a cross-sectional study in Germany

**DOI:** 10.1186/s12889-023-16852-9

**Published:** 2023-10-07

**Authors:** Fiona Kohl, Peter Angerer, Jeannette Weber

**Affiliations:** Institute of Occupational, Social and Environmental Medicine, Centre for Health and Society, Medical Faculty, Heinrich-Heine-University Düsseldorf, Moorenstraße 5, 40225 Düsseldorf, Germany

**Keywords:** Mental health, Workplace, Occupational health, Psychotherapy, Help-seeking behaviour

## Abstract

**Background:**

Psychotherapeutic consultation at work (PT-A) provides employees with mental illnesses or subclinical symptoms a short-term and low-threshold access to psychotherapeutic care. However, practical experience shows that the utilisation rate seems low compared to expected demand. Therefore, this study aimed to identify determinants of utilisation by exploring associations between sociodemographic characteristics, psychological well-being, stigma-related barriers and psychosocial safety climate and the intention to seek PT-A.

**Methods:**

Within a cross-sectional study, 658 participants were recruited via various social media channels in Germany. Participants answered an online questionnaire on potential determinants of (1) intention to seek PT-A in general and of intention to seek PT-A to specifically discuss (2) occupational burden and (3) private burden. Multiple ordinal regression analyses were conducted for the whole study sample and for the subgroups of participants screened positive and negative for current depression.

**Results:**

Lower stigma-related barriers were associated with higher general intention to seek PT-A among all study groups. Lower psychological well-being was associated with higher general intention to seek PT-A and with higher intention to seek PT-A to discuss occupational and private burden, but only so in the subgroup of employees who were screened negative for current depression. Treatment experience was associated with higher intention to seek PT-A for occupational burden among participants screened negative but not among participants screened positive for current depression. No associations were found between age, gender, education or psychosocial safety climate and any variable of intention to seek PT-A.

**Conclusion:**

Those results give an overview on potential determinants for the intention to seek PT-A, but future research with longitudinal designs is needed to confirm that those factors also determine actual utilisation of PT-A. Based on the results, practical implications might include antistigma campaigns and promotion of PT-A adapted to the aims of the consultation.

**Supplementary Information:**

The online version contains supplementary material available at 10.1186/s12889-023-16852-9.

## Introduction

In 2019, it was estimated that 970 million people worldwide were living with a mental disorder [1]. The most common mental disorders include depression and anxiety disorders [1, 2]. In Germany, about every third women and every fourth men develops a mental disorder every year [3]. Mental disorders are furthermore the leading cause of illness-related early retirement [4]. Moreover, in a study with more than 2000 employees, it was found that more than 50% currently suffer from mental distress [5]. Of these participants, all but 0.5% were currently employed [5]. Only a minority of people suffering from mental illnesses receives adequate treatment (e.g. primary care or psychotherapy; [6–8]). In high-income countries like Germany or France, the proportion is only about one third [6, 8, 9].

In addition to stigma-related barriers, people often do not perceive the need for treatment or want to manage the disease themselves [10–12]. However, even when patients seek help, they often encounter major structural barriers including uncertainty about where to find appropriate treatment, long waiting times or insufficient financing (e.g. no health insurance; [10, 13, 14]).

Therefore, services that offer low-threshold and short-term access to psychotherapeutic care are recommended [8, 15, 16]. One approach represents psychotherapeutic consultation at work (PT-A, [17–19]). PT-A aims to reach employees with subclinical symptoms or a mental health diagnosis who are still able to work or are currently on sick leave due to their mental illness. With the help of comprehensive diagnostics and a short term psychotherapeutic intervention, the reasons of psychological distress should be identified and development of a mental illness or its aggravation be prevented [17]. In addition to work-related burden, private burden may also be taken into account within the diagnostics and treatment sessions [18]. The service is offered by psychological or medical mental health care specialists and is free of charge for employees. Funding might be provided by the company or company’s health insurance fund [20], but the employer will not be informed about the employee’s use of the service [17]. There are different implementation approaches so far in terms of location, time and scope of PT-A consultation sessions [17–19, 21]. While the PT-A concept of an ongoing RCT offers up to 17 sessions including psychotherapeutic support during reintegration [17], other concepts offer only one diagnostic session with recommendations for further treatment [19]. Furthermore, the sessions are conducted by arrangement with the companies either on or off the company premises and within or outside working hours [17, 18]. PT-A has certain similarities to the internationally known psychological counselling service, the “Employee assistance programme (EAP)” [22]. Compared to PT-A, EAP can be provided by social workers and other professionals in addition to psychotherapists [23, 24]. Therefore, EAP usually offers psychological counselling but no clinical diagnostics or treatment, and includes other problem areas such as debt, addiction, or family issues [23]. The extent to which EAP or PT-A should be offered depends on the individual care situation. In the German health care system, very long waiting times for a therapy place are common [25]. PT-A thus represents a meaningful complement to standard care. However, low availability of adequate treatment options is also cited as a barrier to utilisation of mental health treatment internationally [26, 27]. Therefore, PT-A could also be applied internationally to gain early access to psychotherapeutic diagnostics and consultation.

Since PT-A is a rather new mental health care model, it is so far only implemented in few companies that cooperate for example with psychosomatic clinics. First evaluations of PT-A show that employees can be reached at an early stage of their mental illness [28]. In addition, male patients were reached to a larger extent compared to standard care [28]. The majority of employees who attended PT-A have sought psychotherapeutic consultation for the first time [18] and continued treatment in standard psychotherapeutic care afterwards [19]. However, initial experience from implementation of PT-A reveals that only one to two% of employees who potentially have access to it actually make us of it [29]. This rate seems low compared to the prevalence of depression, anxiety disorders and other common mental illness in the working population [29]. Comparative utilisation rates can be derived from EAP for which some studies also report low utilisation rates of one to five% [30]. However, other studies report utilisation rates of up to 10% for EAP [31, 32] which are substantially higher than current utilisation rates of PT-A [29].

Research on organisational and personal aspects that inhibit or promote utilisation of PT-A is thus needed. Regarding organisational aspects, a previous study of our workgroup analysed employees’ preferences on organisational aspects of PT-A [33]. Since it is known from previous studies that company size and occupational area are likely to play a role in the implementation of health promotion services at the workplace [34, 35], these aspects were considered in the corresponding study [17]. It was found that conducting PT-A sessions outside company premises (vs. on company premises), in-person (vs. telephone or video call) and combining treatment sessions with diagnostics (vs. diagnostics only) was preferred independently of company size and occupational area [33]. Furthermore, previous research found that employees prefer to discuss occupational burden over private burden during PT-A [29, 33] and that agreement to discuss private burden was even lower among employees of larger compared to middle-sized companies [33]. However, those findings on organisational preferences do not yet explain why utilisation rates of PT-A have been so low so far.

One possible reason for not seeking PT-A might be fear of stigmatisation at the workplace [36, 37]. Employees often avoid disclosing their mental illness out of concern that they will be considered less competent at work or that it will affect their career opportunities [36, 37]. Even though use of PT-A is subject of medical confidentiality and employers and other third parties will not be informed [17], stigma might still be perceived as a barrier of participation. First evidence comes from an EAP study, which demonstrated an association between stigma and intention to seek EAP [24]. Furthermore, it is uncertain in how far internalised stigmatisation might be related to utilisation of PT-A. Stigmatization and discrimination of individuals with mental illnesses can lead affected individuals to internalise such statements themselves and consequently becoming afraid to seek professional treatment [12, 38, 39]. There is evidence that positive attitudes of the management, support of supervisors and colleagues at work towards prevention of work stress and treatment of mental illness can increase the likelihood of seeking psychotherapeutic care [36, 40, 41]. In this context, the “psychosocial safety climate (PSC)” is an instrument to measure such support on companies’ organisational level to protect employees’ mental health [42, 43]. In addition to work- and stigma-related aspects, it is also known that gender, age, education, lower psychological well-being and previous experience of mental health treatment are related to seeking psychotherapeutic treatment [24, 27, 44–47]. According to previous studies, men are less likely to seek psychotherapeutic treatment than women [27]. Furthermore, people who are middle aged [8, 27], with more years of education [27] and lower psychological well-being (e.g. greater severity of depression [16, 27]) showed a higher intention to or actual use of psychotherapeutic treatment. However, it is uncertain whether these results are applicable to PT-A. Within one first investigation of our study group, 155 employees of one German company were questioned and it was found that older employees would be more likely to seek PT-A in a psychologically stressful situation. Furthermore, female gender and higher severity of depression were associated with higher intention to seek PT-A for private burden [29]. To the best of our knowledge, no surveys in Germany or other countries have explored possible associations between sociodemographic characteristics, stigma-related barriers, psychological well-being, psychosocial safety climate and treatment experience with the intention to seek PT-A in a larger sample of employees from different companies.

Understanding aspects that inhibit utilisation is crucial as possible recommendations for further actions can be derived from it (e.g. antistigma campaigns [6]) .Therefore, a previous study has already focused on preferences regarding organisational aspects of implementation and differences between various company sizes and occupational areas regarding those preferences and intention to seek PT-A for different purposes [33]. In the present study, the aim was to exploratively determine the association between sociodemographic characteristics, psychological well-being, stigma-related barriers, psychosocial safety climate and treatment experience with the intention to seek PT-A (1) in general and specifically to discuss (2) occupational burden and (3) private burden. For this purpose, data from the same study sample as the previous study about preferences regarding organisation aspects of implementation was used [33].

The relationship between severity of depression and help-seeking behaviour is widely known [27]. Since depressed employees are also one relevant target group for PT-A, analyses were first conducted in the total study sample and then stratified for participants screened positive and negative for current depression.

## Methods

### Study design and study sample

A cross-sectional design using an online questionnaire was applied for this study. Participants were recruited via advertisements on the social media platforms Facebook, Instagram and LinkedIn between May and August 2021. Recruitment using Facebook and Instagram is known to be a useful way to reach a large target group [48]. Since employees from different companies and company sizes throughout Germany were to be reached, this recruitment method was deemed suitable. However, recruitment using these platforms often leads to an overrepresentation of female and young people [48]. Therefore, LinkedIn was chosen as an additional platform to broaden the possible target group. The advertisements were shown to active users who had specified an age between 18 and 65 and Germany as their place of residence. No further criteria at those platforms were chosen for the selection of users to whom the advertisements were shown. The intention was to ensure that the advertisement was not only shown to users who had previously indicated an interest in mental health or other selective topics. The slogan of the advertisement was “Can we facilitate access to psychotherapeutic service? With a short questionnaire you can help us to find an answer”. The inclusion and exclusion criteria were then presented and it was noted that people with and without previous experience of mental health problems can participate.

Inclusion criteria were age between 18 and 65 years and current employment contract with at least 15 working hours per week. These inclusion criteria were adopted from a PT-A concept, which is currently being evaluated in a RCT [17]. No other inclusion or exclusion criteria were applied. When participants clicked on the advertisement, they were redirected to an external website where they could fill in the questionnaire. Data were collected anonymously. Informed consent was obtained by requiring participants to actively agree to the informed consent form by checking a box. A detailed description of the recruiting process is provided elsewhere [33].

### Study instruments

#### Intention to seek PT-A

As PT-A is only implemented in a few companies so far, studies on actual utilisation would only be possible to a limited extent. Since the intention to seek is known to be a good predictor of actual utilisation of mental health care services [49], the intention to seek PT-A was used in the present study as the outcome measure.

Before participants answered the questions about their intention to seek PT-A, they were given a short description of PT-A. It was explained to the participants that PT-A is offered by the employer, which includes diagnostics and treatment sessions by psychological psychotherapists who are subject to medical confidentiality (also towards the employer). Furthermore, they were informed that participation is free of charge for employees. Subsequently, they were informed that we were interested in their opinion towards PT-A regardless of whether they were currently affected by a mental illness or not.

To measure general intention to seek PT-A, one item with a 5-point Likert scale from 1 = “strongly disagree” to 5 = “strongly agree” was used: “If I were personally in a psychologically stressful situation, I would seek PT-A at my company”. To measure intention to seek PT-A for the purposes to discuss occupational and private burden, participants were asked: “For what purpose would you seek PT-A if you were/are affected by a mental illness?” following two items “private burden” and “occupational burden”. Participants were asked to rate both items on a 5-point Likert scale from 1 = “strongly disagree” to 5 = “strongly agree”. These questions were obtained from a previous survey in a German company, where PT-A was already implemented [29].

#### Stigma-related barriers

In order to identify stigma-related barriers to psychotherapeutic treatment, a German translation of the validated stigma subscale of the questionnaire “Barriers to Access to Care Evaluation scale” (BACE TSS) was used [50]. This subscale consists of twelve items with stigma-related reasons for not using or continuing professional treatment for mental illnesses. Participants were asked whether those reasons would hinder them to use PT-A if they had/would have psychological problems. Response options ranged on a 4-point-Likert scale from 0 = “not at all” to 3 = “a lot” with higher scores indicating a greater barrier. A mean value over all items was calculated [50]. For descriptive purposes, the individual barriers were also evaluated. Mean values of the individual barriers were calculated. In addition, the percentage of those participants who reported the barrier to any extent (score 1, 2 or 3) and the percentage of those who reported the barrier as major barrier (score 3) were determined [50]. Internal consistency was good in this study (Cronbach’s alpha = 0.87).

#### Psychosocial safety climate

A German version of the validated short form of the questionnaire “Psychosocial safety climate” consisting of four items (PSC-4; [42]) was used to collect information on employees perception of the company’s policies, procedures and practices to protect employees mental health and safety [51]. All items were answered on a 5-point-Likert-Scale, ranging from 1 = “Strongly disagree” to 5 = “Strongly agree” [42]. A total score was calculated from the sum of all items ranging from 4 to 20 with higher scores representing a higher degree of perceived psychosocial safety climate. Internal consistency of the PSC-4 was good in this study (Cronbach’s alpha = 0.86).

#### Psychological well-being

Psychological well-being was measured with the validated German version of the 5-item World Health Organization Well-Being Index (WHO-5, [52, 53]). Participants rated on a 6-point-Likert-Scale from 0 = “At no time” to 5 = “All of the time” their well-being within the past two weeks. A sum was calculated from these five items (range 0–25). Then, this sum was multiplied by four. This resulted in a score ranging from 0 = “Worst well-being” to 100 = “Best well-being” [52]. A cut-off score of ≤ 50 was used, which is considered accurate for the screening of depression [52]. Internal consistency was good in this study (Cronbach’s alpha = 0.88).

#### Treatment experience

Participants were asked if they had received a mental diagnosis in the past or if they were currently diagnosed with. If the answer was “yes”, they were further asked whether they had received or were receiving treatment for it. The dichotomous variable “Treatment experience (yes/no)” was formed from these answers. All persons who never received treatment (those with a diagnosis and those without a diagnosis) were assigned “no”. All those who received treatment in the past or currently were assigned to “treatment yes”.

Furthermore, participants were asked whether they were currently unable to work due to a mental disorder.

#### Sociodemographic characteristics

Sociodemographic data included information on age (years), gender (male/female/diverse) and education. Education was categorised according to the International Standard Classification of Education: lower secondary (i.e. school leaving certificate after up to 10 years of primary and secondary education), upper secondary (i.e. school leaving certificate after 11/12–13 years of primary and secondary education with qualification for university entrance) and tertiary education (i.e. university degree including PhD; [54]).

#### Work-related characteristics

Occupational area was classified according to the German classification code of 2010 which consists of ten different areas [55]. Company size was measured with five dimension. The first three dimensions were according to the EU recommendation 2003/361 for micro (1–9 employees), small (10–49 employees) and medium-sized companies (50–249 employees) [56]. Additional, two more dimensions for large-sized companies (250–999 employees and ≥ 1000 employees) were added.

### Data analysis

Statistical analyses were performed using R version 4.1.1 (The R Foundation, Vienna, Austria). For descriptive purposes, means and standard deviations were calculated for continuous variables, frequencies and percentages for categorical variables. Group differences for the variables included in the regression models were calculated between the two subgroups of employees screened positive and negative for current depression. For continuous variables and the variables on intention to seek PT-A, t-tests for independent groups were calculated and chi² tests for categorical variables. All tests were two-tailed using a p-value < 0.05 to indicate statistical significance.

Since the dependent variables used a 5-point Likert response scale and deviated strongly from a normal distribution, multiple ordinal regression models were calculated to identify determinants for intention to seek PT-A. Before conducting these analyses, correlations between the included variables were calculated using Spearman correlation to check for possible multicollinearity [57]. This check is an elementary step in the calculation of ordinal regression, as strong correlations between two or more independent variables can lead to problems in determining which variables contribute to the explanation of the dependent variable. The dependent variables in the different models were intention to seek PT-A (1) in general, (2) intention to seek PT-A to discuss occupational burden and (3) intention to seek PT-A to discuss private burden. The independent variables in each model were socio-demographic data (gender, age and education), psychological well-being, stigma-related barriers, psychosocial safety climate and treatment experience. The three regressions models were calculated for the total study sample and repeated for the subgroups of participants who were screened positive and negative for current depression (WHO-5 ≤ 50). All multiple ordinal regression models were calculated two-tailed using a p-value < 0.05 for indicating statistical significance. Brant tests for parallel regression assumption was used for all ordinal regression analyses to ensure that results can be used for interpretation.

Only complete cases answering all questions on the dependent and independent variables, were considered for analyses. Group comparisons were calculated to determine whether the excluded participants with missing values differed significantly from the study participants. Chi-square tests were calculated for nominal data and t-tests for continuous data.

## Results

### Study population

Recruitment information were provided by Meta with regard to Instagram and Facebook. The advertisement was shown 843,386 times to 422,723 people (63% Instagram and 37% Facebook; 61% women and 39% men). The advertisement was thus shown for at least two times to some users. Half of the users that were reached by the advertisement were between 18 and 24 years old. A total of 2,549 people followed the link to the questionnaire and 1,087 gave written informed consent. A total of 883 participants provided information on the inclusion criteria. After checking inclusion criteria, 848 participants remained. The group of employees with diverse gender was too small for the regression analyses (n = 28) and was therefore excluded. After checking the completeness of the data, 658 participants remained (432 Instagram, 197 Facebook, 12 LinkedIn and 17 “Other”. The option “Other” resulted from the fact that the link to the questionnaire was shared by Facebook, Instagram or LinkedIn users (e.g. to a friend).

The excluded participants (n = 163) mainly accessed the questionnaire by Facebook and were on average older (see Additional file 1). In addition, proportionally more employees with a lower secondary education were excluded due to missing values.

A detailed description of the study population can be found in Table 1. Of the 658 included participants, 70% were screened positive for current depression by WHO-5. Study participants were on average 36 years old and predominantly female (87%). Participants screened positive for current depression had on average more treatment experience, higher rates of stigma-related barriers and lower levels of PSC-4.

On average, all three questions on intention to seek PT-A were rated positively by the total study sample and the subgroup (Table 1). About 80% of participants screened positive and negative for current depression strongly agreed or agreed that they would seek PT-A in general (see Additional file 2). For occupational burden it was about 90% and for private burden about 75%.

The mean score for the stigmatising barriers was 1.09 (Table 1). A detailed list of the individual items can be found in Additional file 3. The highest scores were found for the items “Concern that it might harm my chances when applying for jobs” (mean 1.71), “Concern that people might not take me seriously if they found out I was having professional care” (mean 1.54) and “Concern that I might be seen as weak for having a mental health problem” (mean 1.48).

**Table 1:** Description of study population

Characteristics	Total study sample	Subgroup screened positive for current depression²	Subgroup screened negative for current depression²	Test statistics^7^
	n = 658	n = 463	n = 195	
**Age**, mean (SD, min-max)	36 (12.26, 18–65)	35 (12.31, 18–63)	37 (12.06, 18–65)	t = 1.734; p = 0.084
**Gender, n (**%)				χ^2^ = <0.001; p = 0.997
Female	572 (87)	403 (87)	196 (87)	
Male	86 (13)	60 (13)	26 (13)	
**Health insurance** Statutory, n (%)	622 (95)	439 (95)	183 (95)	χ^2^ = 195; p = 1
**Education**^**1**^, n (%)				
Lower secondary education	112 (17)	86 (19)	26 (13)	χ^2^ = 7.458; p = 0.024
Upper secondary education	241 (37)	178 (38)	63 (32)	
Tertiary education	305 (46)	199 (43)	106 (54)	
**Mental health specific characteristics**				
Previous treatment, n (%)	423 (64)	310 (67)	113 (58)	χ^2^ = 4.463; p = 0.035
Currently diagnosed mental illness, n (%)	314 (48) *(1 Missing)*	251 (54)	63 (32) *(1 Missing)*	
Thereof currently in treatment, n (%)	211 (68) *(3 Missings)*	163 (66) *(3 Missings)*	48 (76)	
Diagnosed mental illness in the past, n (%)	392 (59) *(7 Missings)*	291 (62) *(3 Missings)*	101 (53) *(4 Missings)*	
Thereof in treatment in the past, n (%)	359 (92) *(2 Missings)*	262 (90) *(1 Missing)*	97 (97) *(1 Missing)*	
Incapacity to work due to a mental diagnosis, n (%)	43 (7)	39 (8)	4 (2)	
**Psychological well-being**², mean (SD)	37.8 (20.94)	26.54 (12.23)	64.64 (10.12)	t = 41.36; p < 0.001
**Stigma-related barriers**³, mean (SD)	1.10 (0.70)	1.21 (0.69)	0.83 (0.63)	t = -6.793; p < 0.001
**Psychosocial safety climate**^4^, mean (SD)	9.53 (4.00)	8.81 (3.64)	11.25 (4.31)	t = 6.912; p < 0.001
**Intention to seek psychotherapeutic consultation**				
**1) In general**, mean (SD)	4.26 (1.01)	4.26 (1.00)	4.27 (1.02)	t = 0.145; p = 0.885
**2) Occupational burden**, mean (SD)	4.49 (0.90)	4.47 (0.92)	4.53 (0.84)	t = 0.847; p = 0.397
**3) Private burden**, mean (SD)	4.04 (1.05)	4.06 (1.04)	3.99 (1.09)	t = -0.738; p = 0.461
**Company size (employees)**, n (%)^5^				
1–9	76 (11)	50 (11)	26 (13)	
10–49	136 (21)	92 (20)	44 (23)	
50–249	140 (21)	107 (23)	33 (17)	
250–999	117 (18)	81 (17)	36 (18)	
1000	189 (29)	133 (29)	56 (29)	
**Occupational areas**, n (%)^6^				
Agriculture, forestry, farming and horticulture	4 (1)	2 (1)	2 (6)	
Production of raw materials and goods and manufacturing	19 (3)	14 (3)	5 (3)	
Construction, architecture, surveying and technical building services	15 (2)	13 (3)	2 (6)	
Natural sciences, geography and informatics	50 (8)	35 (8)	15 (8)	
Traffic, logistics, safety and security	26 (4)	19 (4)	7 (4)	
Commercial services, trading, sales, the hotel business and tourism	102 (16)	76 (16)	26 (13)	
Business organisation, accounting, law and administration	102 (16)	73 (16)	29 (15)	
Health care, the social sector, teaching and education	248 (38)	164 (35)	84 (43)	
Philology, literature, humanities, social sciences, economics, media, art, culture and design	76 (12)	54 (12)	22 (11)	
Other	16 (2)	13 (3)	3 (2)	

^1^ Categorised by ISCED 2011 – International Standard Classification of Education [54]; ² measured by “The World Health Organisation - Five Well-Being Index (WHO-5)” with a cut-off value of ≤ 50 to screen for depression (range 0-100) [52]; ³ measured by treatment stigma subscale of “Barriers to Care Evaluation (BACE)” (range 0–36) [50]; ^4^ measured by “4-item Psychosocial safety climate scale (PSC-4)” [42] (range 4–20); ^5^ According to EU recommendation 2003/361 [56]; ^6^ According to the German classification code of 2010 [55]; with two additional dimensions for large-sized companies; ^7^ chi-square tests and two samples t-tests for independent samples comparing the subgroup screened positive with the subgroup screened negative for current depression; n = total number, SD = standard deviation

### Determinants of intention to seek PT-A

Correlations between all study variables are shown in Table 2, suggesting that no multicollinearity exists between the independent variables.

**Table 2:** Correlations between study variables (Spearman correlation)

	1	2	3	4	5	6	7	8	9
1 General intention to seek psychotherapeutic consultation at work	1.00								
2 Intention to seek psychotherapeutic consultation at work for occupational burden	0.25**	1.00							
3 Intention to seek psychotherapeutic consultation at work for private burden	0.18**	0.15**	1.00						
4 Gender	-0.09*	-0.04	0.04	1.00					
5 Age	0.11*	0.09*	-0.04	-0.07	1.00				
6 Education^1^	-0.04	0.03	-0.09*	-0.04	0.08	1.00			
7 Psychological well-being²	-0.02	-0.02	-0.05	-0.02	0.08*	0.11**	1.00		
8 Stigma-related barriers³	-0.28**	-0.05	-0.06	0.05	-0.24**	0.06	-0.28**	1.00	
9 Psychosocial safety climate^4^	-0.02	-0.08*	-0.05	0.03	0.02	0.09*	0.25**	-0.16**	1.00
10 Treatment experience	-0.07	-0.09*	0.01	0.08*	0.01	0.09*	0.17**	-0.03	0.09*

* p-value < 0.05; ** p-value < 0.01; ^1^ Categorised by ISCED 2011 – International Standard Classification of Education [54]; ² measured by “The World Health Organisation - Five Well-Being Index (WHO-5)” with a cut-off value of ≤ 50 to screen for depression [52]; ³ measured by treatment stigma subscale of “Barriers to Care Evaluation (BACE)” [50]; ^4^ measured by “4-item Psychosocial safety climate scale (PSC-4)” [42]

### General intention to seek PT-A

Multiple ordinal regressions were conducted to identify associations of age, gender, education, psychological well-being (WHO-5), stigma-related barriers (BACE TSS), psychosocial safety climate (PSC-4) and previous treatment with general intention to seek PT-A. Results of these multiple ordinal regression analyses can be found in Table 3.

**Table 3:** Multiple ordinal regression predicting general intention to seek psychotherapeutic consultation at work

General intention^5^	Total study sample (n = 658)					Subgroup of participants screened positive for current depression² (n = 463)					Subgroup of participants screened negative for current depression² (n = 195)				
	Beta	Beta 95% CI	OR	OR 95% CI	p-value	Beta	Beta 95% CI	OR	OR 95% CI	p-value	Beta	Beta 95% CI	OR	OR 95% CI	p-value
Age (in years)	0.008	-0.005; 0.022	1.01	0.99; 1.02	0.234	0.007	-0.008; 0.023	1.01	0.99; 1.02	0.356	0.011	-0.016; 0.038	1.01	0.98; 1.04	0.427
Gender (Ref. female)	-0.407	-0.836; -0.028	0.67	0.43; 1.03	0.064	-0.494	-1.005; -0.026	0.61	0.37; 1.03	0.060	-0.237	-1.041; 0.594	0.79	0.35; 1.81	0.568
Education^1^															
Lower education	Ref.					Ref.					Ref.				
Upper education	0.083	-0.377; 0.537	1.09	0.69; 1.71	0.721	0.147	-0.385; 0.670	1.16	0.68; 1.95	0.585	-0.095	-1.049;0.824	0.91	0.35; 2.28	0.842
Tertiary education	-0.042	-0.483; 0.392	0.96	0.62; 1.48	0.851	-0.122	-0.637; 0.383	0.89	0.53; 1.47	0.639	0.168	-0.742;1.041	1.18	0.48; 2.83	0.710
Psychological well-being²	-0.009	-0.017; -0.001	0.99	0.98; 1.00	**0.022**	-0.009	-0.024; 0.006	0.99	0.98; 1.00	0.253	-0.043	-0.073;-0.014	0.96	0.93; 0.99	**0.004**
Stigma-related barriers³	-0.878	-1.121; -0.638	0.42	0.33; 0.53	**< 0.001**	-0.813	-1.089; -0.541	0.44	0.34; 0.58	**< 0.001**	-1.138	-1.673;-0.623	0.32	0.19; 0.54	**< 0.001**
Psychosocial safety climate^4^	-0.008	-0.048; 0.032	0.99	0.95; 1.03	0.685	-0.023	-0.073; 0.028	0.98	0.93; 1.03	0.376	0.003	-0.066;0.071	1.00	0.94; 1.07	0.939
Treatment experience (Ref. yes)	-0.243	-0.559; 0.075	0.79	0.57; 1.08	0.134	-0.163	-0.550; 0.227	0.85	0.58; 1.26	0.412	-0.310	-0.897; 0.279	0.73	0.41; 1.32	0.300

^*1*^*Categorised by ISCED 2011 – International Standard Classification of Education* [54]; *² measured by “The World Health Organisation - Five Well-Being Index (WHO-5)” with a cut-off value of ≤ 50 to screen for depression (range 0-100)* [52]; *³ measured by treatment stigma subscale of “Barriers to Care Evaluation (BACE)” (range 0–36)* [50]; ^*4*^*measured by “4-item Psychosocial safety climate scale (PSC-4)” (range 4–20)* [42];^*5*^*range 1–5; n = number; SE = Standard Error; Ref. = Reference category; CI = confidence interval; p-values indicating statistical significance (< 0.05) are printed in bold*

Lower psychological well-being was associated with higher intention to seek PT-A in the total study sample and in the subgroup screened negative for current depression. Increased levels of perceived stigma-related barriers were associated with lower intention to seek PT-A in the total study sample as well as in both subgroups. Age, gender, education, psychosocial safety climate and treatment experience were not associated with intention to seek PT-A.

### Intention to seek PT-A for occupational burden

Table 4 presents the results of the multiple ordinal regression models to predict intention to seek PT-A for private burden and occupational burden.

**Table 4:** Results of multiple ordinal regression predicting intention to seek psychotherapeutic consultation at work to discuss private and occupational burden

Occupational burden^5^	Total study sample (n = 658					Subgroup of participants screened positive for current depression² (n = 463)					Subgroup of participants screened negative for current depression² (n = 195)				
	Beta	Beta 95% CI	OR	OR 95% CI	p-value	Beta	Beta 95% CI	OR	OR 95% CI	p-value	Beta	95% CI	OR	OR 95% CI	p-value
Age (in years)	0.010	-0.005; 0.024	1.01	0.99; 1.02	0.191	0.012	-0.005; 0.030	1.01	0.99; 1.03	0.173	0.005	-0.021; 0.033	1.01	0.98; 1.03	0.696
Gender (Ref. female)	-0.131	-0.603; 0.361	0.88	0.55; 1.43	0.593	-0.457	-1.006; 0.111	0.63	0.37; 1.12	0.107	0.796	-0.192; 1.927	2.22	0.83; 6.87	0.136
Education^1^															
Lower education	Ref.					Ref.					Ref.				
Upper education	-0.193	-0.689; 0.289	0.82	0.50; 1.33	0.438	-0.046	-0.615; 0.508	0.96	0.54; 1.67	0.872	-0.501	-1.598; 0.510	0.61	0.20; 1.67	0.346
Tertiary education	0.111	-0.375; 0.583	1.12	0.69; 1.79	0.650	0.094	-0.469; 0.642	1.10	0.63; 1.90	0.738	0.138	-0.937; 1.128	1.15	0.39; 3.01	0.791
Psychological well-being²	-0.002	-0.010; 0.007	1.00	0.99; 1.01	0.685	-0.009	-0.026; 0.007	0.99	0.97; 1.01	0.260	-0.029	-0.061; 0.003	0.97	0.94; 1.00	0.073
Stigma-related barriers³	-0.162	-0.415; 0.091	0.85	0.66; 1.10	0.208	-0.053	-0.340; 0.237	0.95	0.71; 1.27	0.720	-0.575	-1.128; -0.033	0.56	0.32; 0.97	**0.039**
Psychosocial safety climate^4^	-0.037	-0.079; 0.005	0.96	0.92; 1.01	0.084	-0.036	-0.089; 0.017	0.96	0.92; 1.01	0.180	-0.068	-0.146; 0.008	0.93	0.86; 1.01	0.084
Treatment experience (Ref. yes)	-0.369	-0.708; -0.028	0.69	0.49; 0.97	**0.033**	-0.188	-0.600; 0.230	0.83	0.55; 1.26	0.374	-0.705	-1.342; -0.075	0.49	0.26; 0.93	**0.029**
**Private burden** ^**5**^															
Age (in years)	-0.007	-0.019; 0.005	0.99	0.98; 1.01	0.272	-0.007	-0.021; 0.008	0.99	0.98; 1.01	0.381	-0.002	-0.025; 0.022	1.00	0.97; 1.02	0.875
Gender (Ref. female)	0.181	-0.237; 0.606	1.20	0.79; 1.83	0.400	0.306	-0.202; 0.827	1.36	0.82; 2.29	0.242	0.029	-0.740; 0.815	1.03	0.48; 2.26	0.942
Education^1^															
Lower education	Ref.					Ref.					Ref.				
Upper education	0.146	-0.282; 0.571	1.16	0.75; 1.77	0.503	0.068	-0.433; 0.565	1.07	0.65; 1.76	0.788	0.320	-0.536; 1.173	1.38	0.58; 3.23	0.461
Tertiary education	-0.219	-0.627; 0.184	0.80	0.53; 1.20	0.288	-0.475	-0.959; 0.001	0.62	0.38; 1.01	0.052	y 0.290898	-0.508; 1.083	1.34	0.60; 2.95	0.472
Psychological well-being²	-0.007	-0.014; 0.001	0.99	0.99; 1.00	0.083	-0.006	-0.021; 0.008	0.99	0.98; 1.01	0.399	-0.048	-0.077; -0.019	0.95	0.93; 0.98	**0.001**
Stigma-related barriers³	-0.250	-0.473; -0.028	0.78	0.62; 0.97	**0.027**	-0.209	-0.463; 0.044	0.81	0.63; 1.05	0.106	-0.430	-0.910; 0.044	0.65	0.40; 1.04	0.077
Psychosocial safety climate^4^	-0.015	-0.053; 0.022	0.98	0.95; 1.02	0.425	-0.012	-0.060; 0.036	0.99	0.94; 1.04	0.612	-0.026	-0.091; 0.038	0.97	0.91; 1.04	0.418
Treatment experience (Ref. yes)	0.098	-0.204; 0.402	1.10	0.82; 1.50	0.525	0.320	-0.056; 0.700	1.38	0.95; 2.01	0.096	-0.219	-0.762; 0.324	0.80	0.47; 1.38	0.429

^*1*^*Categorised by ISCED 2011 – International Standard Classification of Education* [54]; *² measured by “The World Health Organisation - Five Well-Being Index (WHO-5)” with a cut-off value of ≤ 50 to screen for depression (range 0-100)* [52]; *³ measured by treatment stigma subscale of “Barriers to Care Evaluation (BACE)” (range 0–36)* [50]; ^*4*^*measured by “4-item Psychosocial safety climate scale (PSC-4)” (range 4–20)* [42];^*5*^*range 1–5; n = number; SE = Standard Error; CI = confidence interval; Ref. = Reference category; p-values indicating statistical significance (< 0.05) are printed in bold*

Previous treatment experience was associated with a higher intention to seek PT-A for occupational burden in the total study sample and in the subgroup of participants screened negative for current depression. Furthermore, in the subgroup of participants screened negative for current depression, lower psychological well-being and lower stigma-related barriers were associated with higher intention to seek PT-A for occupational burden. No further significant associations were found.

### Intention to seek PT-A for private burden

Higher perceived stigma-related barriers were associated with lower intention to seek PT-A for private burden only in the total study sample. In the subgroup of participants screened negative for current depression, lower psychological well-being was associated with higher intention to seek PT-A for private burden. No significant associations were found for the subgroup of participants screened positive for current depression.

## Discussion

The aim of the present study was to identify determinants of intention to seek PT-A in general and of intention to seek PT-A to specifically discuss private and occupational burden. Overall, the results showed that lower stigma-related barriers, lower psychological well-being were associated with higher general intention to seek PT-A. Those associations were less pronounced for intention to seek PT-A for occupational or private burden. Furthermore, psychological well-being was not significantly associated with the intention to seek PT-A for any purpose in the subgroup of participants screened positive for current depression. Previous treatment experience was associated with a higher intention to seek PT-A for occupational burden among participants screened negative but not among participants screened positive for current depression. No associations were found for age, gender, education and psychosocial safety climate.

The finding that lower psychological well-being is associated with higher intention to seek PT-A is in line with previous studies that found significant associations between psychological well-being [16, 58], severity of depression [27] and help-seeking behaviour for mental health treatment. However, in the present study, psychological well-being was not associated with the intention to seek PT-A among employees who were screened positive for current depression. Those results suggest that intention to seek PT-A increases when psychological well-being declines until the cut-off of depression is reached. Decline of psychological well-being after this cut-off does not seem to explain any further variance in intention to seek PT-A.

Consistent with other studies [12, 27], more perceived stigma-related barriers were also associated with lower intention to seek PT-A in general. However, there were no significant associations between stigma-related barriers and intention to seek PT-A to discuss private or occupational burden among employees screened positive for current depression. A previous study found that stigma related variables are especially related to early stages of help-seeking [59], which could explain why associations are predominantly found among participants screened negative for depression. Furthermore, it might also explain why associations were found with general intention to seek PT-A but not for the intention to seek PT-A for private or occupational burden in the subgroup of participants screened positive for depression. To assess general intention to seek PT-A, participants were asked whether they would seek PT-A in a psychologically stressful situation. To assess intention to seek PT-A to discuss occupational and private burden, however, they were asked, whether they would seek it in the presence of a mental illness and thus a later stage of help-seeking. These results might be particularly relevant for PT-A as it intends to reach also employees before they develop a mental illness or at an early stage of their disease to prevent further progression. For those instances, the results suggest that it is useful to consider stigma-related barriers to increase utilisation of PT-A. However, it should be noted that the intention to seek PT-A in general and to discuss occupational burden was overall rated higher than for private burden. Another possible explanation for the finding that stigma-related barriers were found to be less relevant for intention to seek PT-A to discuss occupational burden among employees screened positive for current depression might be that participants expected different consequences when talking about occupational compared to private burden. For example, a previous systematic review found that one reason for disclosing mental illnesses at the workplace is to achieve job adjustments [60] and participants might have thus expected more personal advantages when using PT-A to discuss occupational burden.

Contrary to previous studies [8, 27], age was not associated with any variables of intention to seek PT-A. This lack of associations may partially be explained by the age structure of the study population. In a comparable study with a broader age distribution, general intention to seek PT-A was higher with increasing age [29]. According to previous studies, especially middle-aged people show higher help-seeking behaviour [8, 27]. As the participants in the present study were, on average, rather young, it cannot be ruled out that this might have had an effect on the results.

In the present study, gender was not associated with the intention to seek PT-A. This may support previous findings that PT-A reaches more men than standard care [28]. Therefore, gender may play a lower role in the use of PT-A. However, in a comparable German survey, female gender was associated with higher intention to seek PT-A to discuss private burden [29]. Male gender was underrepresented in the present study. Lack of associations could therefore also be due to under-representation of male gender.

Education seemed to play a minor role in the intention to seek PT-A in this study. There are controversial findings on the association between education and mental health treatment in previous studies. While some found a significant association with more years of education and a higher help-seeking behaviour towards mental health treatment [27, 45], a German study found the opposite [8]. Higher levels of education seemed to be moderately overrepresented in the present study compared to the general German population [61]. Therefore, a selection bias is possible, which could have influenced the results.

PT-A aims to include work-related aspects in psychotherapeutic sessions, as these often play a subordinate role in standard care [62, 63]. As treatment experience was only associated with the intention to seek PT-A for occupational burden, this may indicate that work-related aspects have not been sufficiently addressed in previous treatments and that participants would therefore like to discuss these aspects in the context of PT-A.

No associations between PSC-4 and the intention to seek PT-A were found in the present study. These results are in contrast to previous studies that found associations between a positive corporate culture regarding mental health treatment and an increase in the likelihood of seeking psychotherapeutic treatment as well as EAP [36, 40, 41, 64]. On the one hand, differences in study results might suggest that corporate culture is related to seeking psychotherapeutic treatment in general and EAP but not to seeking PT-A. On the other hand, discrepancies in study results might be explained by differences in study samples and study instruments. The present study sample came from various companies and occupational areas and thereby differed from these previous studies. In addition, the PSC-4 questionnaire was used in the present study, whereas previous studies mainly used questionnaires specifically adapted to their study populations (e.g. soldiers) [64].

Previous studies embedded factors associated with help-seeking behaviour of individuals with depression in Andersen’s ‘Behavioral Model of Health Services Use’ [27, 58, 65, 66]. This model identifies contextual and personal characteristics related to help-seeking behaviour [66]. These characteristics are further subdivided into predisposing (e.g. gender, age, stigma), enabling (e.g. financing) and need (e.g. severity of depression) factors [66]. The present study was not based on this model, but adds some aspects regarding intention to seek PT-A. As discussed above, this study suggests that typical predisposing factors such as stigma play a role in the intention to seek PT-A, whereas others such as age, gender and education might be less relevant.

### Strengths & limitations

One of the strengths of this study is that the perspective of different target groups are presented. Employees from different companies and occupational areas were included in the survey throughout Germany. This ensures that the results of the present study are transferable to employees from different companies. Furthermore, the study sample consisted of employees screened positive but also negative for current depression. This is another strength of the study, because PT-A intends to reach employees with mental illnesses as well as employees with subclinical symptoms and acute burden such as work-related problems [17].

One limitation is that the questions on the intention to seek PT-A were not derived from validated questionnaires, but were based on a previously conducted study on intention to seek PT-A in one German company [29]. However, since PT-A has not been widely researched, the use of these questions allowed to compare the results of the present study with this comparable previous study [29].

Furthermore, the method used to assess stigma-related barriers needs to be critically discussed [50]. This questionnaire asked participants specifically about which barriers would hinder them to visit PT-A and has thereby directly created a relationship to the intention to seek PT-A. On the one hand, descriptive analyses could thereby show which barriers were specifically important to visit PT-A. On the other hand, overestimation of the association between stigma-related barriers and the intention to seek PT-A seems possible. However, the questionnaire did not differentiate between reasons for seeking PT-A. Our regression analyses could thereby show that reported barriers only seem relevant for using PT-A in general and for private burden but not for occupational burden.

The possibility of a selection bias cannot be ruled out. The recruitment strategy attempted to reach employees with and without depression. However, the majority of participants were screened positive for current depression. This might have been occurred due to the slogan of our advertisements, which potentially attracted more people with current or past mental illnesses. Thus, it is possible that primarily individuals participated who had a higher interest in PT-A due to their personal mental health experience and therefore indicated higher intentions to seek PT-A. However, this aspect was taken into account by including treatment experience in the analyses and by conducting our analyses in a second step for employees screened positive and negative for current depression. Therefore, we were able to show that psychological well-being and treatment experience were related to intention to seek PT-A in the subgroup of participants screened negative but not positive for current depression. This is an important addition to previous knowledge on determinants of seeking mental health treatment.

The study sample was predominantly of young age, female gender and higher education. It was already known in advance that recruitment via Facebook and Instagram leads to this over-representativeness [67]. Therefore, LinkedIn was used as a third social media platform to achieve a more balanced gender and age proportion. However, since only a few people were reached via LinkedIn, this only had a limited influence on the sample distribution. Recruitment via Facebook and LinkedIn showed that half of the people reached were younger than 24 years old and 39% were men. The population was therefore already predominantly young and female. In addition, mainly females followed the link to the questionnaire. It is therefore possible that the advertisement or the study topic appealed less to men than to women. However, male gender is significantly related to lower utilisation of mental health treatment [27] and we cannot exclude the possibility that male participants in our study have higher intention to seek PT-A than non-participants. This could have led to an underestimation of the gender effect. When comparing the study sample with the excluded participants, it was found that mainly people who accessed the questionnaire via Facebook, with lower levels of education as well as higher age had missing values and were therefore excluded. The overrepresentation of higher education in the study population may reflect, on one hand, a known limitation of recruitment via Facebook [67] and, on the other hand, a lower health literacy of people with lower education and thus maybe lower interest in the study topic [68]. Moreover, it might be possible that employees with lower education who participated in our study have higher health literacy and are more willing to use PT-A than non-participants with lower education. This could explain the missing link between education and intention to seek PT-A in our study.

Since no person-specific information were provided from the different social media platforms, it cannot be ruled out that some persons were recruited via Meta and LinkedIn and therefore answered the questionnaire twice. However, due to the high number of users of these platforms and the fact that only twelve people were reached via LinkedIn, the probability of such an event is very low.

The results showed only a few significant associations with intention to seek PT-A. This might have happened because the intention to seek PT-A but not actual utilisation was examined. For instance, it might be possible that a high proportion of male participants in the present study indicated a high intention to seek PT-A, but would not actually make use of it in practice. Thus, the intention to seek PT-A would not show significant associations between gender and intention to seek PT-A, while actual utilisation would represent this effect. Even though it is known that intention to seek is a good predictor of actual utilisation [44, 49], it would be more relevant to examine actual utilisation. Since PT-A is still a fairly new concept and therefore not yet established on a broad scale in the companies and the utilisation rate in the companies is very low so far, actual utilisation could not be used as an outcome at this time point. Therefore, future studies should focus on actual utilisation in order to verify whether the associations found in the present study also apply to actual utilisation.

In the present study, the intention to seek PT-A was assessed with different multiple ordinal regressions. Because of these multiple tests, there is a risk of alpha error accumulation, which means that some significant associations may have been found by chance.

Due to the cross-sectional design of this study, only conclusions about associations can be drawn but not on causal relationships. However, the results might inform researchers about which determinants are reasonable to consider in future longitudinal studies.

### Implications

It is uncertain whether all relevant aspects for prediction of intention to seek PT-A were taken into account in the present study as the different ordinal regression models found only few significant associations. One explanation might include the possibility that some relevant determinants for intention to seek PT-A were missed in this study. For instance, low perceived need for treatment has been found as a reason for not seeking help for mental illness in previous studies [7, 10, 69]. This aspect was not considered in the present study. The majority of participants stated that they would seek PT-A in a psychologically stressful situation or to discuss private or occupational burden in the presence of a mental illness. However, it is uncertain to what extend these participants would actually perceive the need for PT-A and whether they would actually make use of it. Although intention to seek mental health treatment is known as a good predictor for actual utilisation [44, 49], no conclusions on actual utilisation can be drawn with the results of the present study. Therefore, perceived need of PT-A and actual utilisation needs to be taken into account in future studies. Thereby, more representative study samples regarding age, gender and education should be taken into account.

Furthermore, enabling factors including financial aspects as proposed by the Behavioral Model of Health Services Use [27] were not considered in this study, since PT-A is supposed to be free of charge for employees. Nevertheless, indirect effects are conceivable since waiting times for therapy places in standard care are shorter for people with private health insurance than for people with statutory health insurance [70, 71]. In Germany, especially high-income earners are given the opportunity to join private health insurance. However, the extent to which this factor might play a role cannot be determined as the number of participants in private health insurance was too low in the present study.

Working hours might be another important work-related aspect, because fewer working hours might lead to greater flexibility in arranging therapy appointments in care as usual and might thus influence the need to seek help by PT-A. Since this aspect was not considered in the present study, future studies should take it into account.

While other studies found that a positive corporate culture towards transparency and treatment of mental illness can increase the likelihood of seeking psychotherapeutic care [36, 40, 41], no associations were found between intention to seek PT-A and PSC-4. Therefore, further studies are needed to support or disprove this aspect. It might be possible that this association would be significant in a more representative study population. However, it is possible that the PSC-4 does not adequately reflect this aspect, thus other instruments might be necessary to examine which work-related aspects predict intention or actual use of PT-A. Social support might be one relevant aspect. Social support is known as an enabling aspect for seeking treatment and covers private (e.g. friends; [27, 58]) as well work (e.g. colleagues) relationships [40].

The extent to which a migration background or language barriers might play a role was not considered in the present study. However, as it is known from previous studies that migration background can be related to a lower use of psychotherapeutic treatment [72, 73], this aspect should be considered in future studies.

Practical implementation might consider significant predictors of intention to seek PT-A. As supported by previous studies [27, 40, 59], stigma-related barriers were associated with lower intention to seek PTA in general for all study groups. Furthermore, descriptive results of the stigma-related barriers demonstrated that the two work-related aspects (“Concern that it might harm my chances when applying for jobs“ and “Concern about what people at work might think, say, or do“) were among the highest rated barriers (see Additional file 1). There is already evidence that workplace awareness and anti-stigma campaigns can successfully reduce perceived stigma at work and improve supportive behaviour towards people with mental-health problems as well as help-seeking behaviour for mental health issues [74, 75]. Therefore, similar campaigns could accompany implementation of PT-A to increase utilisation by employees.

Stigma-related barriers were not associated with the intention to seek PT-A for occupational burden among employees screened positive for current depression. Therefore, one might discuss whether work-related aspects could be given primary focus in the promotion of PT-A (e.g. in a slogan). However, it should be considered that work-related problems cannot usually be thematised completely separated from private issues during consultation [18]. Therefore, employees should be informed about content and possibilities of PT-A to ensure that all relevant target groups are addressed.

The high intention to seek PT-A found in the present as well as in a previous study is in contrast to previous utilisation rates [29]. Therefore, one might discuss whether employees are not aware of the consultation offered by their company. This is supported by a previous study on EAP utilisation among male employees, which found that the employees were partially unaware of the offer or needed further information on it [76]. At the same time, studies could show that broad advertisement by the employer as well as a targeted recommendation by occupational physicians had a positive effect on utilisation [19, 41]. Specific suggestions for advertising EAP were made in previous studies [45, 76]. For example, the language used in advertisements could be adapted to different target groups (e.g. men; [45, 76]). Furthermore, the use of an ambassador to promote the offer was suggested [76]. An ambassador could be a successful manager of the company who has already participated in EAP himself and actively promotes it [76].

## Conclusion

The present study investigated the determinants of intention to seek PT-A for different purposes among employees screened positive and negative for current depression. The results of this study suggest that higher stigma-related barriers, higher psychological well-being and no treatment experience are associated with lower intention to seek PT-A. However, these associations were dependent on (1) whether participants would seek it in general or specifically to discuss occupational or private burden and (2) whether participants were screened positive or negative for current depression. No significant associations were found for age, gender, education and psychosocial safety climate. Future studies on actual utilisation of PT-A with a study population that is more representative of the working population (e.g. longitudinal studies) are needed to verify these results. Thereby, other possible determinants for utilisation of PT-A (e.g. social support) should be taken into account. Practical recommendations based on the results include anti-stigma campaigns and advertising PT-A adapted to the purposes of the consultation.

### Electronic supplementary material

Below is the link to the electronic supplementary material.

Supplementary Material 1

Supplementary Material 2

Supplementary Material 3

## Data Availability

The datasets used and/or analysed in this study can be handed out in anonymised form. Please contact the author Fiona Kohl (fiona.kohl@uni-duesseldorf.de) in this regard.

## List of Abbreviations

BACE: TSS Barriers to Access to Care Treatment Stigma Subscale
EAP: employee assistance program
OR: Odds Ratio
PSC-4: 4-item Psychosocial Safety Climate questionnaire
SE: Standard Error
WHO-5: The World Health Organisation – Five Well-Being Index
