# Supplementary material for: Determinants of the intention to seek psychotherapeutic consultation at work - a cross-sectional study in Germany
Source: BMC Public Health. 2023 Oct 7;23:1945. doi: 10.1186/s12889-023-16852-9 (PMC10559521; doi:10.1186/s12889-023-16852-9)
Supplement: Supplementary file 1 — Supplementary Material 1 [file 12889_2023_16852_MOESM1_ESM.docx]

**Determinants of the intention to seek psychotherapeutic consultation at work - a cross-sectional study in Germany**

Fiona Kohl^1^, Peter Angerer^1^, Jeannette Weber^1^

^1^ Institute of Occupational, Social and Environmental Medicine, Centre for Health and Society, Medical Faculty, Heinrich-Heine-University Düsseldorf, Moorenstraße 5, 40225 Düsseldorf, Germany

Corresponding author: Fiona Kohl, Institute of Occupational, Social and Environmental Medicine, Centre for Health and Society, Medical Faculty, Heinrich-Heine-University Düsseldorf, Moorenstraße 5, 40225 Düsseldorf, Germany, Email: Fiona.Kohl@hhu.de

**Additional file 2 – Descriptive results of intention to seek psychotherapeutic consultation at work**

Table 1 Descriptive Results of Intention to seek psychotherapeutic consultation at work

|  | **Total study sample (n = 658)** | | | | | | | **Subgroup of participants screened positive for current depression² (n = 463)** | | | | | | | **Subgroup of participants screened negative for current depression² (n = 195)** | | | | | |
| --- | --- | --- | --- | --- | --- | --- | --- | --- | --- | --- | --- | --- | --- | --- | --- | --- | --- | --- | --- | --- |
|  | Strongly disagree | Disagree | | Partly-Party | Agree | Strongly Agree | | Strongly disagree | Disagree | | Partly-Party | Agree | Strongly Agree | | Strongly disagree | Disagree | Partly-Party | | Agree | Strongly Agree |
| **Intention to seek psychotherapeutic consultation** | | | | | | |  | | |  | | | |  | | | |  | | |
| **1) In general** | | |  | | | |  | | |  | | | |  | | | |  | | |
| Total | 15 (2) | 34 (5) | | 78 (12) | 167 (25) | 364 (55) | | 9 (2) | 27 (6) | | 53 (12) | 120 (26) | 254 (55) | | 6 (3) | 7 (4) | 25 (13) | | 47 (24) | 110 (56) |
| Female | 11 (2) | 29 (5) | | 64 (11) | 142 (25) | 326 (57) | | 5 (1) | 25 (6) | | 42 (10) | 103 (26) | 228 (57) | | 6 (4) | 4 (2) | 22 (13) | | 39 (23) | 98 (58) |
| Male | 4 (5) | 5 (6) | | 14 (16) | 25 (29) | 38 (44) | | 4 (7) | 2 (3) | | 11 (18) | 17 (28) | 26 (43) | | 0 (0) | 3 (12) | 3 (12) | | 8 (31) | 12 (46) |
| **2) Occupational burden** | | |  | | | |  | | |  | | | |  | | | |  | | |
| Total | 11 (2) | 24 (4) | | 44 (7) | 132 (20) | 447 (68) | | 8 (2) | 19 (4) | | 33 (7) | 90 (19) | 313 (68) | | 3 (2) | 5 (3) | 11 (6) | | 42 (22) | 134 (69) |
| Female | 8 (1) | 20 (3) | | 35 (6) | 118 (21) | 391 (68) | | 5 (1) | 16 (4) | | 25 (6) | 80 (20) | 277 (69) | | 3 (2) | 4 (2) | 10 (6) | | 38 (22) | 114 (67) |
| Male | 3 (3) | 4 (5) | | 9 (10) | 14 (16) | 57 (65) | | 3 (5) | 3 (5) | | 8 (13) | 10 (17) | 36 (60) | | 0 (0) | 1 (4) | 1 (4) | | 4 (15) | 20 (77) |
| **3) Private burden** | | |  | | | |  | | |  | | | |  | | | |  | | |
| Total | 18 (27) | 45 (7) | | 108 (16) | 207 (31) | 280 (43) | | 12 (3) | 27 (6) | | 82 (18) | 141 (30) | 201 (43) | | 6 (3) | 18 (9) | 26 (13) | | 66 (34) | 79 (41) |
| Female | 17 (3) | 41 (7) | | 94 (16) | 179 (31) | 241 (42) | | 11 (3) | 26 (6) | | 72 (18) | 123 (31) | 171 (42) | | 6 (4) | 15 (9) | 22 (13) | | 56 (33) | 70 (41) |
| Male | 1 (1) | 4 (5) | | 14 (16) | 28 (33) | 39 (45) | | 1 (2) | 1 (2) | | 10 (17) | 18 (30) | 30 (50) | | 0 (0) | 3 (12) | 4 (15) | | 10 (38) | 9 (35) |

Data are given as numbers (n) and percent (%) ^1^ Measured by “The World Health Organisation - Five Well-Being Index (WHO-5)” with a cut-off value of ≤ 50 to screen for depression (Range 0 – 100) [1]

1. Topp CW, Østergaard SD, Søndergaard S, Bech P: **The WHO-5 Well-Being Index: a systematic review of the literature**. *Psychother Psychosom* 2015, **84**(3):167-176.
