# Supplementary material for: Determinants of the intention to seek psychotherapeutic consultation at work - a cross-sectional study in Germany
Source: BMC Public Health. 2023 Oct 7;23:1945. doi: 10.1186/s12889-023-16852-9 (PMC10559521; doi:10.1186/s12889-023-16852-9)
Supplement: Supplementary file 2 — Supplementary Material 2 [file 12889_2023_16852_MOESM2_ESM.docx]

**Determinants of the intention to seek psychotherapeutic consultation at work - a cross-sectional study in Germany**

Fiona Kohl^1^, Peter Angerer^1^, Jeannette Weber^1^

^1^ Institute of Occupational, Social and Environmental Medicine, Centre for Health and Society, Medical Faculty, Heinrich-Heine-University Düsseldorf, Moorenstraße 5, 40225 Düsseldorf, Germany

Corresponding author: Fiona Kohl, Institute of Occupational, Social and Environmental Medicine, Centre for Health and Society, Medical Faculty, Heinrich-Heine-University Düsseldorf, Moorenstraße 5, 40225 Düsseldorf, Germany, Email: Fiona.Kohl@hhu.de

**Additional file 1 – Comparison between included study participants and excluded participants due to missing values**

Table 1 Comparison between study population (n = 658) and excluded participants with missing values (n = 163)

|  | Included participants | Excluded participants | Test statistics |
| --- | --- | --- | --- |
| **Access via,** n (%) |  |  |  |
| Instagram | 432 (66) | 42 (26) | 🗶² = 87.743, p = < 0.001 |
| Facebook | 197 (30) | 97 (60) |  |
| LinkedIn | 12 (2) | 9 (6) |  |
| Other | 17 (3) | 13 (8) |  |
| **Age, mean** | 35.73 | 42.27 | t = 5.756, p = < 0.001 |
| **Gender,** n (%) |  |  | 🗶²= 0.020, p = 0.887 |
| Female | 572 (87) | 143 (88) |  |
| Male | 86 (13) | 20 (12) |  |
| **Education^1^,** n (%) |  |  | 🗶² = 8.841, p = 0.012 |
| Lower secondary education | 112 (17) | 42 (26) |  |
| Upper secondary education | 241 (37) | 44 (27) |  |
| Tertiary education | 305 (46) | 77 (47) |  |
| **Health insurance,** n (%) |  |  | 🗶² = 0.166, p = 0.684 |
| Statutory | 622 (95) | 156 (96) |  |
| Private | 36 (5) | 7 (4) |  |
| **Psychological well-being**²**,**  mean (SD) | 37.83 | 40.58  (n = 159) | t = 1.401, p = 0.163 |

*n = number; SD = standard deviation; x² = chi-squared test; t = t-test for independent samples; p = p-value; ^1^ Categorised by ISCED 2011 – International Standard Classification of Education [1]; ² measured by “The World Health Organisation - Five Well-Being Index (WHO-5) [2]”*

1. UNESCO Institute for Statistics: **International standard classification of education**; 2011.

2. Topp CW, Østergaard SD, Søndergaard S, Bech P: **The WHO-5 Well-Being Index: a systematic review of the literature**. *Psychother Psychosom* 2015, **84**(3):167-176.
