# Supplementary material for: Determinants of the intention to seek psychotherapeutic consultation at work - a cross-sectional study in Germany
Source: BMC Public Health. 2023 Oct 7;23:1945. doi: 10.1186/s12889-023-16852-9 (PMC10559521; doi:10.1186/s12889-023-16852-9)
Supplement: Supplementary file 3 — Supplementary Material 3 [file 12889_2023_16852_MOESM3_ESM.docx]

**Determinants of the intention to seek psychotherapeutic consultation at work - a cross-sectional study in Germany**

Fiona Kohl^1^, Peter Angerer^1^, Jeannette Weber^1^

^1^ Institute of Occupational, Social and Environmental Medicine, Centre for Health and Society, Medical Faculty, Heinrich-Heine-University Düsseldorf, Moorenstraße 5, 40225 Düsseldorf, Germany

Corresponding author: Fiona Kohl, Institute of Occupational, Social and Environmental Medicine, Centre for Health and Society, Medical Faculty, Heinrich-Heine-University Düsseldorf, Moorenstraße 5, 40225 Düsseldorf, Germany, Email: Fiona.Kohl@hhu.de

**Additional file 3 – Results of stigma-related barriers**

Table 1 Results of the questionnaire „ Barriers to Access to Care Evaluation scale (BACE)” n = 658 [1]

| **Stigma-related barrier items^1^** | **Reporting item as a barrier to any degree** % (n)^2^ | **Reporting item as a major barrier** % (n)^3^ | **Total** (n) | **Mean (SD)*** |
| --- | --- | --- | --- | --- |
| Concern that I might be seen as weak for having a mental health problem | 81 (533) | 18 (118) | 658 | 1.48 (0.99) |
| Concern that it might harm my chances when applying for jobs | 82 (518) | 30 (191) | 630 | 1.71 (1.08) |
| Concern about what my family might think, say, do, or feel | 37 (242) | 5 (35) | 658 | 0.57 (0.87) |
| Feeling embarrassed or ashamed | 67 (440) | 9 (57) | 658 | 1.06 (0.95) |
| Concern that I might be seen as ‘crazy’ | 56 (366) | 11 (75) | 658 | 0.96 (1.04) |
| Concern that I might be seen as a bad parent | 47 (80) | 8 (13) | 172 | 0.74 (0.95) |
| Concern that people I know might find out | 49 (324) | 7 (49) | 658 | 0.77 (0.94) |
| Concern that people might not take me seriously if they found out I was having professional care | 78 (515) | 23 (151) | 658 | 1.54 (1.07) |
| Not wanting a mental health problem to be on my medical records | 64 (420) | 18 (121) | 658 | 1.21 (1.12) |
| Concern that my children may be taken into  care or that I may lose access or custody | 42 (61) | 15 (22) | 144 | 0.82 (1.12) |
| Concern about what my friends might think, say, or do | 32 (209) | 2 (14) | 658 | 0.43 (0.72) |
| Concern about what people at work might think, say, or do | 76 (498) | 17 (109) | 646 | 1.39 (1.02) |

*^1^Response options ranged on a 4-point-Likert scale from 0 = “not at all” to 3 = “a lot” with higher scores indicating a greater barrier; ^2^ Responses scored 1,2 or 3 on the scale; ^3^ Responses scored 3 on the scale; n = number; SD = standard deviation*

**References**

1. Clement S, Brohan E, Jeffery D, Henderson C, Hatch SL, Thornicroft G. Development and psychometric properties the Barriers to Access to Care Evaluation scale (BACE) related to people with mental ill health. BMC Psychiatry. 2012;12(1); doi: 10.1186/1471-244X-12-36.
